# CMD-05, a novel promising clinical anti-diabetic drug candidate, in vivo and vitro studies

**DOI:** 10.1038/srep46628

**Published:** 2017-04-13

**Authors:** Jie Ma, Huan Li, Xiangnan Hu, Lu Yang, Qi Chen, Congli Hu, Zhihao Chen, Xiaoyan Tian, Yang Yang, Ying Luo, Run Gan, Junqing Yang

**Affiliations:** 1 Department of Pharmacology, Chongqing Medical University, the Key Laboratory of Biochemistry and Molecular Pharmacology, Chongqing 400016, China

## Abstract

Dipeptidyl peptidase IV (DPP-IV) inhibitor has been expected to be a new class of anti-diabetic agent. The present study was designed to characterize the pharmacological profiles of CMD-05, a novel DPP-IV inhibitor discovered in our laboratory, *in vitro* and *in vivo*. The IC_50_ of CMD-05 on DPP-IV inhibitory activity was approximately 12 nM while vildagliptin was 3.5 nM *in vitro*. In diabetes rat model established by high fat diet/low dose streptozotocin, CMD-05 inhibited DPP-IV activity, significantly improved glucose tolerance, increased GLP-1 and insulin levels in plasma. Long-term administration of CMD-05 decreased HbA1c and TG levels and improved the islet function without significantly effect on body weight. Furthermore, CMD-05 reduced INS-1 cell apoptosis and increased GLP-1 secretion in NCI-H716. After oral administration, CMD-05 reached peak concentration at 30 min with half-life of 288 minutes and the inhibitory rate of DPP-IV greater than 50% lasted for 15 h. In fasted normal rats, CMD-05 didn’t cause significant hypoglycemia. CMD-05 had a lower cytotoxicity than vildagliptin *in vitro* and its maximum tolerance dose in mice is beyond 2000 mg/kg. These results indicated that CMD-05 has similar activity with vildagliptin *in vivo* and has a much longer half-life and lower cytotoxicity than vildagliptin.

Type 2 diabetes mellitus (T2DM) is a metabolic disease that develops when insulin secretion from islet beta cells were not sufficient^1^,^2^,^3^,^4^, it is strongly associated with obesity and insulin resistance^5^, as well as defects in pancreatic β cell mass and function^6^. Streptozotocin (STZ) is widely used as known as representative in the field of diabetes research that is particularly toxic to pancreatic β cells in mammals. A lot of versions of the HFD/STZ models have been reported with different STZ strategies and more and more researchers prefer to consider it as T2DM model rather than T1DM^7^,^8^,^9^,^10^,^11^. Reported in the reference, T1DM patients have lost 70–90% of their beta cell mass^12^, and the β cells fraction is approximately 10% in T1DM rats induced by high dose STZ^13^. In obese and lean patients with T2DM, 63 and 41% in relative β cell volume compared with non-diabetic cases^6^ and HFD/STZ rats had ~40% reduction of total β cell mass compared with non-diabetic rats^14^, which indicated that the experiment model of diabetic induced by HFD combined use with low dose STZ is consistent with the pathological changes of clinical T2DM patients.

In clinical, various anti-diabetic drugs including metformin, sulfonylureas, thiazolidinediones and insulin *etc.* are currently available to treat T2DM, but the strategies have limited in long-term efficacy and tolerability in the nature progressive of the disease^15^,^16^. In addition, several side effects have been reported ranged from hypoglycemia, weight gain and nausea^17^. Thus, it is necessary to develop safe and effective therapeutic agents which would improve glucose homeostasis^18^.

The incretin hormone, glucagon-like peptide 1 (GLP-1), is released from L cells in the intestine in response to food intake, which plays an important role in regulating postprandial blood glucose levels in a glucose-dependent manner^19^,^20^,^21^. In addition, GLP-1 increases β cell mass^22^, inhibits the apoptosis of β cell^23^, and stimulates β cell proliferation, survival and neogenesis in the pancreas^22^,^24^. However, the activated GLP-1 is rapidly degraded by enzyme dipeptidyl peptidase-IV (DPP-IV), resulting in its circulating half-life is only 1–2 min^25^,^26^. Thus, DPP-IV inhibitors, which can prevent GLP-1 from the proteolytic degradation and enhance glucose-dependent insulin secretion from pancreatic β cells, have been expected to become a novel approach for the treatment of T2DM with a minimal risk of hypoglycemia and weight gain^25^,^27^,^28^.

Various selective DPP-IV inhibitors especially adamantane-based DPP-IV inhibitor have been developed and marketed as therapeutic agents for T2DM including vildagliptin in EU in 2007 (Galvus, Novartis)^29^,^30^, and saxagliptin in the US in 2009 (Onglyza, BMS)^31^ are now used in many countries as monotherapies or in combination with other glucose-lowering agents. Oral vildagliptin and saxagliptin has shown higher bioavailability but shorter half-life. The application of vildagliptin and saxagliptin would affected the patients with renal insufficiency due to they are excreted by means of glomerular. In addition, the most common side effect with vildagliptin (seen in between 1 and 10 patients in 100) is dizziness^32^. Vildagliptin has been associated with liver problems as a case of elevated aminotransferase reported^33^. Therefore, it is urgent to find a safe and effective DPP-IV inhibitor with wide application.

CMD-05, which is a novel, orally available, adamantane-based DPP-IV inhibitor discovered in our laboratory, shows a unique chemical structure that is a kind of vildagliptin analogue. Therefore, in this study we used vildagliptin as positive control. The aim of the present studies is to characterize the pharmacological profile of CMD-05 with regard to the flowing points: (1) Test the pharmacodynamics characteristics in rats and cells. (2) Test the pharmacokinetics characteristics in rats after oral and intravenous injection of CMD-05. (3) Evaluate the safety profiles in cells, mice and rats. In the present study, we found that as an effective, less toxic and absolutely novel anti-diabetic candidate drug, CMD-05 plays an important role in improving hyperglycemia, which suggested that CMD-05 is expected to be a novel DPP-IV inhibitor.

## Results

### Inhibitory effects of CMD-05 on recombinant human DPP-IV activity

**Figure 1:**
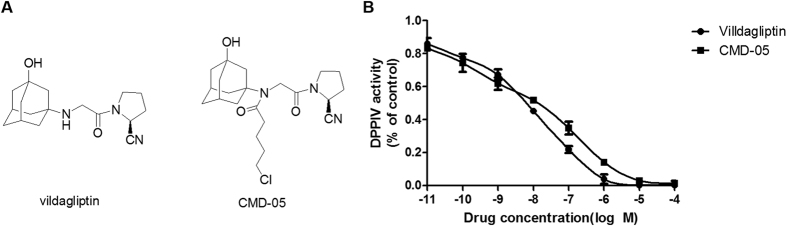
Structure and DPP-IV inhibition of CMD-05. The structures of vildagliptin and CMD-05 (**A**). The inhibitory effect of CMD-05 on recombinant human DPP-IV (**B**). Vildagliptin and CMD-05 inhibited DPP-IV activity with an IC_50_ of approximately 3.5 nM and 12 nM, respectively. Data are expressed as the mean ± SD of three determinations.

As shown in Fig. 1B, the inhibitory of CMD-05 on DPP-IV activity was in a concentration dependent manner and the inhibitory IC_50_ of CMD-05 was 12 nM under our experimental conditions. As an internal control, the inhibitory IC_50_ of vildagliptin was 3.5 nM under our experimental condition, which was close to 3 nM in the description.

### Effect of CMD-05 on oral glucose tolerance of model rats

**Figure 2:**
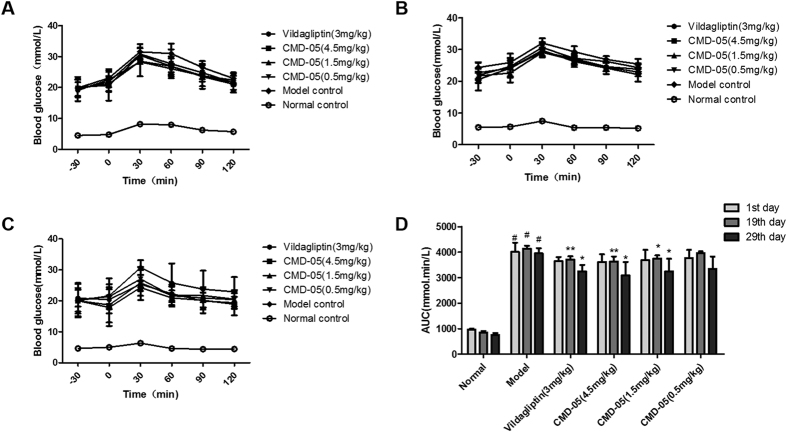
Effects of CMD-05 on changes of blood glucose in rats subjected to oral glucose tolerance test. Changes in blood glucose levels of oral glucose tolerance test on the 1st day (**A**), the 19th day (**B**) and the 29th day (**C**), respectively. Changes in the area under the curve (AUC) of the blood glucose in oral glucose tolerance test on the 1st day, the 19th day and the 29th day (**D**). Results are expressed as means ± SD of five individual animals. ^#^P < 0.01 *vs* normal group. **P < 0.01, *P < 0.05 *vs* model group, respectively.

On the 1st day (Fig. 2A), the 19th day (Fig. 2B) and the 29th day (Fig. 2C), blood glucose reached the peak concentration at 30 min after oral glucose, and this hyperglycemia was maintained until 120 min in model rats. CMD-05 (4.5 mg/kg) significantly decreased the blood glucose levels and the percentage reduction was 9.7%, 12.2% and 21.9%, respectively. And the percentage reduction of CMD-05 (1.5 mg/kg) was 8.0%, 9.22% and 17.8%, respectively. Furthermore, CMD-05 led to a significant decrease of the AUC (area under the blood glucose curve) (Fig. 2D). Meanwhile, vildagliptin (3 mg/kg) significantly reduced the blood glucose levels by 8.9%, 10.1% and 17.8% and the AUC values in vildagliptin-treated groups were also decreased in a time-dependent manner.

### Effect of CMD-05 on plasma DPP-IV activity in model rats

**Figure 3:**
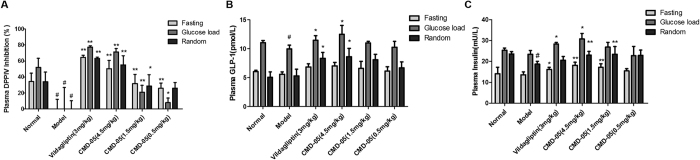
Effects of CMD-05 on DPP-IV activity, GLP-1 levels and insulin levels in fasted, glucose load and random plasma of rats. DPP-IV activity (**A**). GLP-1 levels (**B**). Insulin levels (**C**). Results are expressed as means ± SD of five individual animals. ^#^P < 0.01 *vs* normal group. **P < 0.01, *P < 0.05 *vs* model group, respectively.

An obvious dose-dependently inhibitory of DPP-IV was measured in fasted, glucose load and random rat plasma (Fig. 3A). In fasted plasma, the DPP-IV inhibition rate was about 60%, 50%, 30 and 20% in the group of vildagliptin (3 mg/kg), CMD-05 (4.5 mg/kg), CMD-05 (1.5 mg/kg) and CMD-05 (0.5 mg/kg), respectively. In glucose load plasma, the DPP-IV inhibition was about 80%, 70%, 20 and 10%, respectively. In random plasma, the DPP-IV inhibition was about 60%, 55%, 30 and 20%, respectively. The potency of CMD-05 (4.5 mg/kg) was not apparently different with vildagliptin (Fig. 3A).

### Effect of CMD-05 on GLP-1 and insulin levels in model rats

An obvious increase levels of plasma GLP-1 (Fig. 3B) and insulin (Fig. 3C) were measured in fasted, glucose load and random rat plasma. CMD-05 (4.5 mg/kg) significantly increased the levels of GLP-1 in glucose load and random plasma. Vildagliptin played a similar role to CMD-05 (4.5 mg/kg). Administration of CMD-05 (1.5 and 4.5 mg/kg) significantly increased insulin levels in fasted, glucose load and random plasma of model rats. Meanwhile, vildagliptin also significantly increased insulin levels in fasted, glucose load plasma.

### Effect of CMD-05 on HbA1c level in model rats

**Figure 4:**
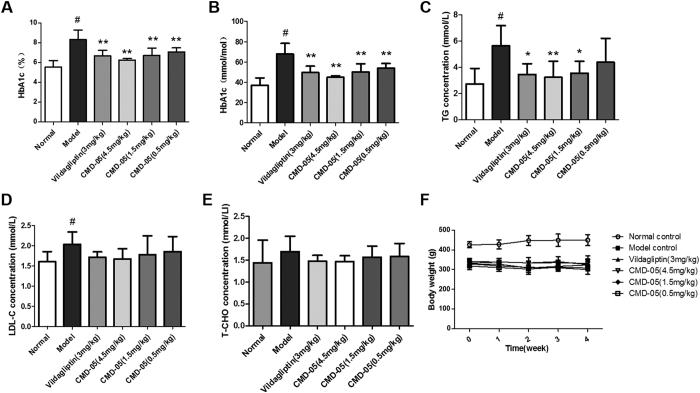
Effects of CMD-05 on the levels of HbA1c, TG, LDL-C and T-CHO and the changes of body weight in model rats. HbA1c (**A** and **B**), TG (**C**), LDL-C (**D**), T-CHO (**E**), body weight (**F**). Results are expressed as means ± SD of five animals. ^#^P < 0.01 *vs* normal group. **P < 0.01, *P < 0.05 *vs* model group, respectively.

Figure 4(A) (Different units) showed the levels of HbA1c in blood. Compared with normal rats, the level of HbA1c in model was significantly increased, and compared with model rats, it was significantly decreased in CMD-05-treated groups. The level of HbA1c was reduced by 1.0%, 2.2% and 2.0% in group of CMD-05 (1.5 mg/kg), CMD-05 (4.5 mg/kg) and vildagliptin, respectively.

### Effect of CMD-05 on TG, LDL-C and T-CHO levels in model rats

As shown in Fig. 4(C) and (E), the increased plasma levels of TG and LDL-C in model rats was significantly suppressed by vildagliptin and CMD-05. The TG level in model rats was reduced by 38.79%, 42.67%, 37.07% and 22.41% in vildagliptin (3 mg/kg)-, CMD-05 (4.5 mg/kg)-, CMD-05 (1.5 mg/kg)- and CMD-05 (0.5 mg/kg)-treated group, respectively. The LDL-C level was reduced by 15.97%, 17.91%, 12.60 and 9%, respectively. After administration of CMD-05 (4.5 mg/kg) continuously for 30 days, the plasma lipids levels in model rats were decreased to normal level.

### Changes in body weight of rats

As shown in Fig. 4F, compared with normal rats, the body weights of model rats were decreased. There was no significant difference in body weights between CMD-05- and vildagliptin-treated groups and model group. There was also no significant difference between before and after administration of CMD-05 and vildagliptin.

### Morphologic observation on islet of rats

**Figure 5:**
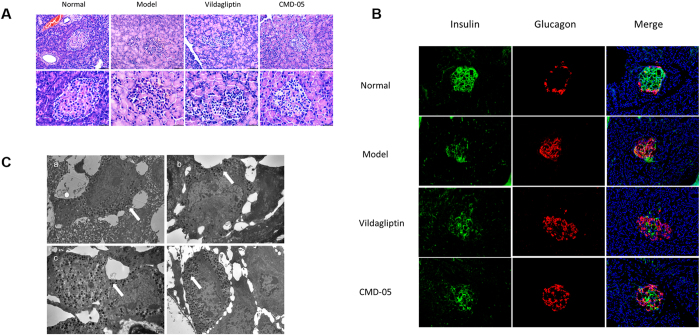
Morphologic observation on islet of rats. (**A**) HE staining, 200×, 400×, respectively. (**B**) Double immunofluorescent staining for insulin (green), glucagon (red) and DAPI (blue), 400×. (**C**) Effect of CMD-05 on the secretory granules of islet cell in rats, 12000×, Scale bar = 1 μm.

Morphology of pancreatic islets was examined by HE staining. In model rats, the morphology and structure of islets showed incomplete and damage. The administration of CMD-05 and vildagliptin could prevent islet from damage. Furthermore, the protective effect of CMD-05 (4.5 mg/kg) on islet was a little bit better than vildagliptin (Fig. 5A).

Insulin and glucagon double staining showed that the distribution of β cells was loose and α-cells were disordered in model rats. And in the CMD-05- and vildagliptin- treated groups, the distribution of β cells were obviously improved and the mass of α-cells were mildly reduced (Fig. 5B).

### Effect of CMD-05 on the secretory granules in islet cell of rats

Figure 5C showed the ultrastructure changes in β cells of rats. Beta cells in islet contains a number of secretory granules which has a space between the core and the membrane and distributes diffusely in the cytoplasm. The granule consists of a central core and an external single-layered membrane usually with moderate homogenous or slightly heterogeneous electro density. Transmission electron microscopic analysis of β cells in pancreatic islet showed no pathological alterations in normal rats. In our study, the secretory granules in β cells of model rats (b) were obviously fewer than that of normal rats (a). And compared with the model, secretory granules in β cells of vildagliptin- (c) and CMD-05- (d) treated rats were obviously increased.

### Effect of CMD-05 on apoptosis and proliferation in INS-1 cell

**Figure 6:**
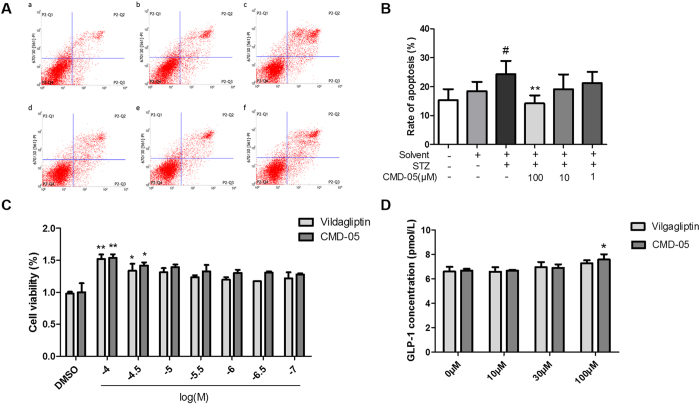
Effect of CMD-05 on the apoptosis and proliferation of INS-1cell and the GLP-1 secretion in NCI-H716 cells. (**A**) Determination of apoptotic cells with flow cytometry. (**B**) The percentage of apoptotic cells was shown after analysis by flow cytometer. Data were expressed as mean ± SD of three determinations. ^#^P < 0.01 solvent group. **P < 0.01 *vs* STZ group. (**C**) Effect of CMD-05 on proliferation ofINS-1 cells measured by a CCK-8 assay. Data are expressed as the mean ± SD of three determinations. **P < 0.01, *P < 0.05 *vs* DMSO group, respectively. (**D**) Effect of CMD-05 on GLP-1 secretion in NCI-H716 cells. Data are means ± SD (n = 4), *P < 0.05 *vs* DMSO group.

In INS-1 cells, STZ led to significantly apoptosis, while incubation with CMD-05 resulted in reduce of apoptosis and in a concentration dependent manner (Fig. 6A and B).

The effect of CMD-05 on proliferation activity in INS-1 cells was evaluated by the CCK-8 assay. As shown in Fig. 6C, compared with the solvent group (DMSO), cells viabilities were increased in the group treated with CMD-05 (1 × 10^−4^ and 3 × 10^−5^ M) and vildagliptin.

### Effect of CMD-05 on GLP-1 secretion in NCI-716 cells

In NCI-716 cells, CMD-05 significantly increased GLP-1 secretion in a concentration-dependent manner, which was increased from 6.6 ± 0.14 pmol/L to 7.5 ± 0.44 pmol/L in concentration of 100 μM CMD-05. And the GLP-1 levels in vildagliptin groups increased slightly without significance compared with solvent group (Fig. 6D).

### Pharmacokinetics studies

**Figure 7:**
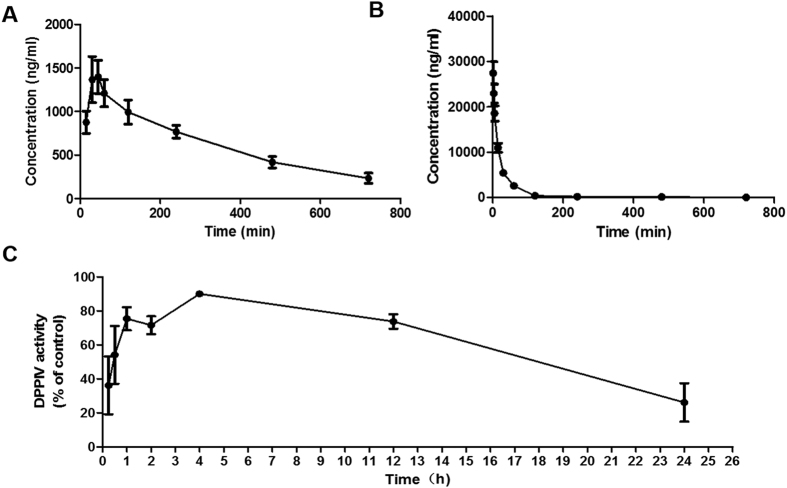
Pharmacokinetics of CMD−05 in rats. (**A**) Concentration-time curve following single oral administration of 50 mg/kg CMD-05. (**B**) Concentration-time curve following single intravenous administration of 50 mg/kg CMD-05. (**C**) Changes of DPP-IV activity following single oral administration of 4.5 mg/kg CMD-05. Data are expressed as the mean ± SD of four individual animals.

The plasma concentration of CMD-05 in rats were measured after a single intravenous administration (50 mg/kg) and single oral administrations (50 mg/kg), respectively. As showed in Fig. 7A, after oral administration of CMD-05, the plasma levels reach the peak concentration at 30–45 min, and then gradually decreased. Half-life of CMD-05 is 287.9 ± 26.2 min, which is considerably greater than vildagliptin according to the ref. 34. The absolute bioavailability of CMD-05 in rats was 84.5%. Furthermore, as showed in Fig. 7C the inhibition of plasma DPP-IV activity reached maximum (about 90%) at 4 hours after the dose and the inhibition has been greater than 50% lasted for at least 15 hours.

### Measure of cytotoxicity of CMD-05

**Figure 8:**
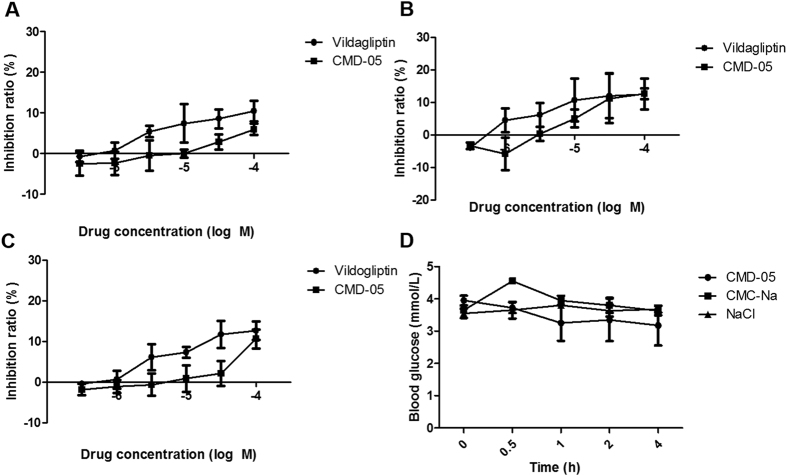
Cytotoxicity of CMD-05 and acute hypoglycemia effect of CMD-05. 293 cells (**A**), HT22 cells (**B**), LO-2 cells (**C**). Data are expressed as the mean ± SD of three determinations. (**D**) Acute hypoglycemia of CMD-05 on rats. Data are expressed as the mean ± SD of three individual animals.

The effects of vildagliptin and CMD-05 on cell viabilities of 293 (Fig. 8A), HT22 (Fig. 8B) and LO-2 (Fig. 8C) cells were evaluated by the CCK-8 assay. In 293 cells, the different concentration of CMD-05 range from 3 × 10^−6^ to 1 × 10^−5^ M did not induce significant cytotoxicity, 1 × 10^−4^ M of CMD-05 slightly reduced cell viability whereas the inhibition ratio of vildagliptin was beyond 10% at concentration of 1 × 10^−4^ M. In HT22 cells, the different concentration of CMD-05 range from 3 × 10^−6^ to 3 × 10^−5^ M did not induce significant cytotoxicity whereas the inhibition ratio of vildagliptin was beyond 10% at concentration of 1 × 10^−5^ M. In LO-2 cells, there was no detectable toxic effect of CMD-05 at a concentration of 1 × 10^−4^ M, while the inhibition ratio of vildagliptin was beyond 10% at concentration of 1 × 10^−5^ M.

### Acute hypoglycemia effect and acute toxicity of CMD-05

In the acute hypoglycemic test, 50 mg/kg of CMD-05 was orally administered after fasting 12 h in normal SD rats, and glucose levels were measured at 0, 0.5, 1, 2 and 4 h after administration. As shown in Fig. 8D, 50 mg/kg of CMD-05 had little effects on glucose levels. The results suggested CMD-05 could not have the potential side effect of hypoglycemia.

In the acute toxicity test, CMD-05 exhibited low acute toxicity which had no toxicological signs at a single oral dose of 2000 mg/kg in mice.

## Discussion

Diabetes mellitus especially T2DM is becoming the main severe public health problem affecting the physical and mental health of human^35^. As a kind of drug depend on hormone secretion, compared to other traditional drugs, DPP-IV inhibitor not only has a good hypoglycemic effect, but also has good safety and tolerance, which is widely recognized in clinical^36^. The inhibition of DPP-IV could lead to the action of GLP-1^27^,^37^,^38^, which is known to induce glucose-dependent stimulation of insulin secretion and suppress glucagon secretion. Furthermore, numerous studies showed that GLP-1 increases β cell mass, proliferation and neogenesis^39^,^40^,^41^ and inhibits β cell apoptosis and necrosis^23^,^39^. However, the vildagliptin^42^ and saxagliptin^31^ has emerged some negative effects such as dizziness or liver problem, so it is necessary to develop a novel DPP-IV inhibitor.

In the present study, we have synthesized the compound CMD-05 which is a kind of vildagliptin analogues. Studies showed that CMD-05 inhibited Recombinant Human DPP-IV, with IC_50_ values of approximately 12 nM which is about three times of vildagliptin (3.5 nM). This suggested that CMD-05 has meaningful activity *in vitro*, which is worth further study. Therefore, we evaluated the efficacy of CMD-05 from aspects of pharmacodynamics, pharmacokinetics and safety.

In our investigation, the experimental model of T2DM rats was induced by high fat diet combined with low dose of STZ intraperitoneal injection (30 mg/kg) to evaluate the efficacy of CMD-05. A persistent high-fat and high-carbohydrate diet is account for accumulated glucolipotoxicity, resulting in β cells dysfunction, insulin resistance, and a low-dose STZ may partially damage islet cells and generate mild inflammation around islets, which could accelerate the progress of β cells dysfunction and insulin deficiency^11^,^43^,^44^,^45^. In our models, the β cells reduced about 40% compared with normal control observed by immunofluorescence, and the secretion of insulin decreased about 30% compared with normal control in basic plasma, which is consistent with the clinical patient and animal models according to the reports^6^,^14^. These indicators confirmed that our model was a model of T2DM.

The T2DM rats were continuous treated with CMD-05 and vildagliptin for 30 days. On the 1st day, 19th days and 29th days after administration of CMD-05, the oral glucose tolerance of the model rats was improved, and the role of CMD-05 (4.5 mg/kg) was better than vildagliptin (3 mg/kg). Furthermore, CMD-05 dose-dependently inhibited the activity of DPP-IV in fasted, glucose load and random plasma of model rats significantly. CMD-05 (4.5 mg/kg) had a similar DPP-IV inhibition rate to vildagliptin. CMD-05 showed a significant increase of the level of GLP-1 in glucose load and random plasma and significantly increased insulin levels in fasted, glucose load and random plasma. These results suggested that CMD-05 could inhibit the activity of DPP-IV, reduce the decomposition of GLP-1 and increase insulin secretion, which play a positive role in decreasing blood glucose. In addition, long-term administration of CMD-05 could decrease the HbA1c, TG and LDL-C levels, improve the destruction of islet morphology and function, and increase the number of secretory granules in β cells. Interestingly, the inhibitory activity of CMD-05 *in vitro* is less than vildagliptin, but hypoglycemic activity of CMD-05 *in vivo* is slightly greater than vildagliptin. This phenomenon suggested that CMD-05 may play a role in reducing blood glucose by means of other than the inhibition of DPP-IV. However, this need to be confirmed by further experiments.

According to reports, INS-1 cell line is widely used in the growth and survival of islet cells^39^,^46^. Some studies have reported that DPP-IV inhibitors could promote cell survival in INS-1 cells exposed to STZ^13^. In our experiment, INS-1 cells were also served as apoptosis model induced by STZ to observe the anti-apoptotic effect of CMD-5. Results showed a significantly anti-apoptotic effect of CMD-5 which was consistent with the results *in vivo*. Furthermore, CMD-05 can promote the proliferation of INS-1 cells in a concentration dependent manner which also was consistent with the protective effect CMD-05 on islet β cells *in vivo*. GLP-1 is secreted from intestinal endocrine L-cells^47^. The NCI-H716 cell line was widely used as a unique human model to study the regulation of GLP-1 secretion^48^. In our experiments, NCI-H716 cells were used to further investigate the effect of CMD-05 on regulation of GLP-1 release. Our studies showed that CMD-05 may stimulate GLP-1 secretion from the NCI-H716 cells in a dose-dependent manner and that this effect was slightly better than vildagliptin. These results indicated that CMD-05 not only could inhibit the degradation of islet β cells but also contribute to the release of GLP-1.

In the present experiment of pharmacokinetics, CMD-05 was well absorbed in healthy male rats after oral administration, with a peak plasma concentration of CMD-05 at 30 to 45 mins. CMD-05 displayed an oral bioavailability of 84.5% in healthy rats, and its pharmacokinetics was not affected by food. The half-life of CMD-05 is about 288 minutes, which is longer than vildagliptin. Considering the rapid absorption similar with vildagliptin, favorable inhibition of DPP-IV activity with maximum inhibition about 90% and plasma DPP-IV inhibition lasted for 15 h in rats, CMD-05 can be designed as a once-daily dosing regimen.

In cytotoxicity studies, CMD-05 exhibited low toxicity in 293, HT22 and LO-2 cells which is lower than vildagliptin. In single dose acute toxicity study, CMD-05 exhibited low acute toxicity which had no toxicological signs at a single oral dose of 2000 mg/kg in mice. CMD-05 also exerted little effects on glucose levels of normal rats.

In conclusion, the present preclinical studies indicated that CMD-05 is a potent DPP-IV inhibitor with good pharmacokinetic characteristics and safety. As an analogue of vildagliptin, CMD-05 is better than vildagliptin in some respects. The anti-diabetic mechanism of CMD-05 is to inhibit the activity of DPP-IV and then to inhibit the decomposition of GLP-1. However, that CMD-05 decreases islet cell apoptosis and increases GLP-1 release also might contribute to the anti-diabetic mechanism. Furthermore, the effects of CMD-05 on different diabetic animal models of diabetes should be performed and the hypoglycemic mechanism of CMD-05 should be further clarified.

## Methods

### Materials

CMD-05 was synthesized in our laboratories (Fig. 1A) (Patent apply number: 201610818878.5). Purity of CMD-05 was 98.9% which was determined by high performance liquid chromatography (HPLC). Vildagliptin was of analytical grade *(Aladdin, China)* for study *in vitro*, and vildagliptin *(Galvus, Novartis)* was used *in vivo*.

### Recombinant human DPP-IV activity assay

DPP-IV inhibition assay was carried out using either 5 ng purified recombinant human DPP-IV (rhDPP-IV) *(R&D systems, USA)*. Recombinant DPP-IV was mixed with ddH_2_O in an assay buffer (25 mM Tris-HCl, 0.2 M NaCl, 0.1% BSA, pH 7.3). Briefly, the assay was carried out by incubating 50 μl of the rhDPP-IV with or without different concentrations of CMD-05 in 1% DMSO for 15 min at 25 °C in 96-well black plate, followed by the addition of 25 μl 2.0 mM Gly-Pro-MCA *(AnaSpec, USA)* (100 μM final) in a buffer. The vehicle comprised 1% DMSO in place of test compounds. The fluorescence intensity of 7-amino-4-methyl-coumarin (AMC) generated from Gly-Pro-MCA was measured using an automated microplate reader at 353 nm excitation and 442 nm emission. The fluorescence intensity of AMC corresponded to DPP-IV activity.

### Animals

Sprague-Dawley (SD) rats were housed in the barrier housing facility, and it has in keeping with national standard “Laboratory Animal-Requirements of Environment and Housing Facilities”. The care of laboratory animal and the animal experimental operation have conforming to “Chongqing Administration Rule of Laboratory Animal”. The experimental procedures were approved by the animal laboratory administrative center and the institutional ethics committee of Chongqing Medical University (License number: SYXK YU 2012-0001) and also in accordance with the National Institutes of Health guidelines. The rats were kept in controlled conditions of temperature (24 ± 2 °C), relative humidity (60 ± 10%) and 12/12 h light/dark cycle (light from 08:00 am to 08:00 pm).

To establish model of T2DM, male rats (80–100 g, 9-week-old)^49^ were fed high fat diet (HFD) (20% sugar, 10% lard, 10% egg yolk and 60% basal feed) after a week of normal diet. After 4 weeks, rats were injected once with low-dose streptozotocin *(Solarbio, China)* (STZ, 30 mg/kg i.p) to induce partial insulin deficiency, and then continuously fed HFD for 4 weeks after injection of STZ. Then the model rats were oral administrated drugs for 30 days. The body weights were recorded once a week.

For acute hypoglycemia test, male rats (150–250 g) were used. For acute toxicity test, Kunming mice with half males and half females (18–22 g) were used.

### Oral glucose tolerance test (OGTT)

On the 1st day, 19th days and 29th days of treatment, oral glucose tolerance tests for model rats were carried out. The 12h-fasted rats were orally pre-dosed with vildagliptin (3 mg/kg), CMD-05 (4.5 mg/kg), CMD-05 (1.5 mg/kg), CMD-05 (0.5 mg/kg) (The dose of CMD-05 4.5 mg/kg is equal to vildagliptin 3 mg/kg according to the same molar concentration) and vehicle (0.25% CMC-Na) at −30 min, followed by orally administrated with glucose (2 g/kg) after 30 min. Blood samples were taken from the orbital venous at −30 and 0 min before glucose administration and at 30, 60, 90 and 120 min after glucose administration. The blood glucose levels were tested using Johnson one touch Ultra Test Strips on Johnson Performa blood glucose meter.

### Biochemical assays

Oral glucose load, fasted and random blood samples were collected from rats on the 28th days, the 29th days and the last of the treatment of drugs, respectively. Plasma levels of insulin and GLP-1 were measured by ELISA kits *(YuanYe, China)* according to the manufacturer’s recommendation. The levels of HbA1c, Triglycerides (TG), total cholesterol (TC) and low density lipoprotein cholesterol (LDL-C) in blood samples were measured by commercial assay kits *(JianCheng, Nanjing, China)* according to the manufacturer’s directions.

### Plasma DPP-IV activity determination

Oral glucose load, fasted and random blood samples was collected to measure DPP-IV activity at the 28th days, the 29th days and the last treatment of the CMD-05. DPP-IV enzyme assay was carried out according to a published method^4^. The enzyme reaction was initiated by adding 5 mM Gly-Pro-AMC as a substrate. After 25 min of incubation, fluorescence of AMC released by the reaction was measured using a spectrofluorometer at an excitation wavelength of 353 nm and emission wavelength of 442 nm.

### Immunohistochemistry and transmission electron microscopy

HE staining on paraffin section was performed as described in reports^11^. In brief, paraffin section were rinsed with PBS, fixed with 4% paraformaldehyde (PFA) for 30 min and then washed by PBS. The pancreas tissue were stained with Hematoxylin-Eosin, afterwards, they were dehydrated in alcohols. Morphological changes of the pancreas tissue were observed under an optical microscope *(Olympus, Japan)* after mounted by neutral resins.

Pancreas sections (8 μm) were cut in a cryostat and collected on superfrost microscope slides. The slides were stored at −20 °C till analysis. Immunofluorescent staining was used for detection of insulin (β cells) *(1:100, Anti-Insulin antibody, Abcam)*, glucagon (α cells) *(1:200, Anti-glucagon, Abcam)*. Briefly, the slides were blocked in 5% normal goat serum for 1 h in a humidified chamber. Slides were exposed to primary antibodies at a dilution of 1:200 for insulin and glucagon in antibody diluting solution at 4 °C overnight, washed in PBS, and exposed to appropriate secondary antibodies linked to the fluorescent probes (Goat Anti-Guinea pig IgG/FITC and Alexa Fluor 594-conjugated Goat Anti-Rabbit IgG) *(Bioss and Proteintech, China)* in antibody diluting solution for 1 h and DAPI *(Beyotime, China)* for 5 min in the dark. After washed by PBS, slides were mounted with a glycerol mounting medium. Images were collected using a fluorescent microscope *(Nikon, Japan)*.

For transmission electron microscopy, about 1 mm^3^ tissue specimens were obtained from the same pancreas region of the control and experimental groups of rats, and fixed overnight in 2% glutaraldehyde (pH7.3) in 0.1 M phosphate buffer saline at 4 °C. Images of pancreatic islets were captured from random tissue sections using a transmission electron microscope.

### Determination of INS-1 cells activity *in vitro*

The rat insulinoma cell line INS-1 (832/13) was obtained from the Department of endocrinology at 1st affiliated hospital of Chongqing medical university. Cells were cultured in RPMI 1640 *(Gibco, USA)* (11 mmol/l glucose, 100 units/ml penicillin, 100 g/ml streptomycin, 10 mmol/l HEPES pH 7.4) supplemented with 10% FBS *(Gibco, USA)*, 2 mmol/l L-glutamine, 1 mmol/l pyruvate, and 50 μmol/l β-mercaptoethanol. Cells were treated with CMD-05 (1 × 10^−7^, 3 × 10^−7^, 1 × 10^−6^, 3 × 10^−6^, 1 × 10^−5^, 3 × 10^−5^, 1 × 10^−4^M) and vildagliptin (1 × 10^−7^, 3 × 10^−7^, 1 × 10^−6^, 3 × 10^−6^, 1 × 10^−5^, 3 × 10^−5^, 1 × 10^−4^ M) and then cultured in 96-well flat bottomed microliter plates for 24 h. 10 μl of CCK-8 was then added and incubated at 37 °C and 5% CO_2_ for 1.5 h and optical difference (OD) was read at 460 nm with microplate reader. The OD value represents the proliferation activity.

The INS cells were seeded into 6-well culture plates at 3 × 10^4^ cells per ml and incubated at 37 °C for 24 h. Then supernatants were replaced by medium contained with vildagliptin (1 μM, 10 μM, 100 μM) and CMD-05(1 μM, 10 μM, 100 μM). After incubated for 36 h, apoptosis was assessed by flow cytometry using the Annexin V-FITC/ propidium iodide (Annexin V/PI) apoptosis detection kit according to the manufacturer’s protocol.

### GLP-1 secretion measure in NCI-H716 cells

Human enteroendocrine NCI-H716 cells were maintained in suspension culture as described by the American Type Culture Collection guideline (ATCC). Two days before the experiments, cells were seeded into 12-well culture plates coated with Matrigel at 1 × 10^6^ cells per well. Cells were incubated in RPMI 1640 containing 10% FBS at 37 °C for 48 h. On the day of the experiments, supernatants were replaced by KRB (115 mM of NaCl, 4.71 mM of KCl, 1.28 mM of CaCl_2_, 1.2 mM of KH_2_PO_4_, 1.2 mM of MgSO_4_, 10 mM NaHCO_3_, 0.3% BSA, PH 7.2) contained vildagliptin (10 μM, 30 μM, 100 μM) and CMD-05 (10 μM, 30 μM, 100 μM). After incubated for 2 h, GLP-1 concentrations from the supernatants were measured by ELISA kits *(YuanYe, China)*.

### Pharmacokinetics characteristic studies

Male SD rats (200–220 g) were fasted overnight to administration of CMD-05 intravenously *via* tail vein (50 mg/kg, n = 4) or by oral gavage (50 mg/kg, n = 4). Blood samples of 0.3 ml were collected from the orbital venous at 1 min, 3 min, 5 min, 15 min, 30 min, 60 min, 120 min, 240 min, 480 min, 720 min after tail vein injection of CMD-05 and at 15 min, 30 min, 45 min, 60 min, 120 min, 240 min, 480 min, 720 min after oral of CMD-05. Plasma was obtained after centrifugation. The concentrations of CMD-05 in plasma were determined by HPLC method. The pharmacokinetic parameters of CMD-05 in the rats were analyzed by DAS2 software.

Blood samples were collected after administration of CMD-05 (4.5 mg/kg) at 15 min, 30 min, 1 h, 2 h, 4 h, 12 h, and 24 h to measure the DPP-IV activity in fasting normal rats, the concrete steps refer to the preceding.

### Cell toxicity assessment and acute toxicity of mice

The toxicities of CMD-05 on HT22, 293 and LD-05 cell lines was assessed using Cell Counting Kit-8 (CCK-8) *(Biosharp, China).* Briefly, the cells were plated on 96-well plates. Following treatments of CMD-05 (1 × 10^−6^, 3 × 10^−6^, 1 × 10^−5^, 3 × 10^−5^, 1 × 10^−4^, 3 × 10^−4^ M) and vildagliptin (1 × 10^−6^, 3 × 10^−6^, 1 × 10^−5^, 3 × 10^−5^, 1 × 10^−4^, 3 × 10^−4^ M) for 12 h, CCK-8 was added to each well and incubated at 37 °C for 1.5 h. The absorbance at 450 nM was measured using a microplate spectrophotometer.

Acute toxicological signs of included activities, nervous system response, autonomic nervous system response and death of mice were observed after a single oral dose of 2000 mg/kg.

### Acute hypoglycemia assay

Normal SD rats were used to determine the potential effect of CMD-05 on hypoglycemia. In the assay, CMD-05 (50 mg/kg, n = 9) was orally administered to rats after 12 h fasting. Glucose levels were measured from tail blood at 0, 1, 2, 4, 6 and 8 h using Johnson one touch Ultra Test Strips on Johnson Performa blood glucose meter.

### Statistical analysis

Data are presented as mean ± SD. Statistical analysis was carried out using SPSS Statistics software (Version 17.0) and data were analyzed by performing one-way Analysis of Variance (ANOVA) followed by Dunnett type multiple comparison test. P value less than 0.05 was considered statistically significant.

## Additional Information

**How to cite this article:** Ma, J. *et al*. CMD-05, a novel promising clinical anti-diabetic drug candidate, in vivo and vitro studies. *Sci. Rep.* **7**, 46628; doi: 10.1038/srep46628 (2017).

**Publisher's note:** Springer Nature remains neutral with regard to jurisdictional claims in published maps and institutional affiliations.
